# Urinary cell microRNA-based prognostic classifier for non-muscle invasive bladder cancer

**DOI:** 10.18632/oncotarget.15315

**Published:** 2017-02-14

**Authors:** Mercedes Ingelmo-Torres, Juan José Lozano, Laura Izquierdo, Albert Carrion, Meritxell Costa, Lidia Gómez, María José Ribal, Antonio Alcaraz, Lourdes Mengual

**Affiliations:** ^1^ Laboratory and Department of Urology, Hospital Clínic de Barcelona, Centre de Recerca Biomèdica CELLEX, Institut d’Investigacions Biomèdiques August Pi i Sunyer (IDIBAPS), Universitat de Barcelona, Barcelona, Spain; ^2^ CIBERehd, Plataforma de Bioinformática, Centro de Investigación Biomédica en Red de Enfermedades Hepáticas y Digestivas, Hospital Clínic de Barcelona, Barcelona, Spain

**Keywords:** biomarkers, bladder cancer, microRNA, tumour progression, urine

## Abstract

Current prognostic tools for non-muscle invasive bladder cancer (NMIBC) do not have enough discriminative capacity to predict the risk of tumour progression. This study aimed to identify urinary cell microRNAs that may be useful as non-invasive predictive biomarkers of tumour progression in NMIBC patients. To this end, 210 urine samples from NMIBC patients were included in the study. RNA was extracted from urinary cells and expression of 8 microRNAs, previously described by our group, was analysed by quantitative PCR. A tumour progression predicting model was developed by Cox regression analysis and validated by bootstrapping. Regression analysis identified miR-140-5p and miR-92a-3p as independent predictors of tumour progression. The risk score derived from the model containing these two microRNAs was able to discriminate between two groups with a highly significant different probability of tumour progression (HR, 5.204; p<0.001) which was maintained when patients were stratified according to tumour risk. The algorithm was also able to identify two groups with different cancer-specific survival (HR, 3.879; p=0.021). Although the data needs to be externally validated, miRNA analysis in urine appears to be a valuable prognostic tool in NMIBC patients.

## INTRODUCTION

Non-muscle invasive bladder cancers (NMIBC) have a 1- and 5-year disease-progression rate of up to 17% and 45%, respectively [1]. The current most important clinical prognosticators in NMIBC patients, according to the EORTC risk tables, are stage T1, high grade (HG) and presence of concomitant carcinoma *in situ* (CIS) [2]. Nevertheless, these prognostic factors do not have sufficient discriminative capacity to predict, at the patient level, the risk of tumour progression. Progressive patients deserve careful attention, particularly because therapeutic decisions change when patients are likely to progress. Many efforts have been made to develop biomarkers for assessing aggressiveness and for predicting the prognosis of NMIBC patients (reviewed in [3]), but to date none of the identified biomarkers is being used in the clinical setting due to their limited value. Thus, there is a clear need for accurate and reliable predictive biomarkers that can distinguish progressive from non-progressive NMIBC.

In recent years it has become apparent that gene expression is controlled post-transcriptionally by microRNAs (miRNAs), short (21–23 nt) non-coding RNAs which bind to specific mRNAs. Experimental work has shown that miRNAs are dysregulated in most cancer types, including bladder cancer (BC) [4–7], and have demonstrated significant diagnostic and prognostic values in different malignancies [8–11]. In the case of BC, the assessment of the levels of these aberrantly expressed miRNAs in urine samples appears to be a promising approach to identify diagnostic and prognostic biomarkers in a non-invasive way. Urine is a particular source of such biomarkers since tumour cells are in direct contact with it, it is collected noninvasively and miRNA have shown high stability and easy detectability in urine [12]. Using a case-control design, we have shown that urinary miRNA profiles vary significantly between urines from control and BC patients and between high and low grade tumours [13]. Since we have previously found a urinary miRNA signature representative of tumour aggressiveness, we decided to explore the hypothesis that urinary miRNAs may be useful as predictive biomarkers of progression in NMIBC patients. We thus compared the urinary miRNA profiles from individuals with NMIBC who progressed with those who did not progress. Subsequently, we developed a prognostic urinary miRNA signature that was further internally cross-validated.

## RESULTS

### Characteristics of the dataset

A total of 210 consecutive urinary samples from BC patients with NMIBC subjected to transurethral resection of the bladder (TURB) were included (Table 1). Overall, 22 (11%) patients developed tumour progression and 42 patients (20%) died, 10 of them (5%) due to BC, after a mean follow up of 47 months. The mean time to tumour progression and cancer-specific survival was 19 and 23 months, respectively.

**Table 1:** Clinicohistopathologic features of the bladder cancer patients

VARIABLES	
**Gender_N (%)**	
Male	157 (75)
Female	53 (25)
**Age_years**	
Mean	71
Range	37-94
**Tumour stage and grade_N (%)^1^**	
Tis	15 (7)
Ta LG	77 (37)
Ta LG+CIS	1
Ta HG	22 (10)
Ta HG+CIS	3 (1)
T1 LG	1
T1 LG+CIS	15 (7)
T1 HG	59 (28)
T1 HG+CIS	17 (8)
**Tumour focality_N (%)**	
Unifocal	108 (51)
Multifocal	102 (49)
**Tumour diameter (%)**	
≤3 cm	170 (81)
>3 cm	40 (19)
**Prior recurrence (%)**	
Primary tumours	104 (50)
Recurrent tumours	106 (50)
**BCG treatment (%)**	98 (47)
**TOTAL**	**210**

^1^The grade and stage of the tumours were determined according to WHO criteria [38] and TNM classification [39], respectively.

Abbreviations: CIS or Tis, Carcinoma *in situ*; LG, Low grade; HG, High grade; BCG, Bacillus Calmette-Guerin

The clinical classifiers for progression, stage, grade, presence of CIS, multiplicity and tumour size were analysed in both progressive and non-progressive patients. Stage T1 and HG tumours were identified in 44% and 54% of non-progressive and in 41% and 68% of progressive patients, respectively. Concomitant CIS was found in 18% of progressive and non-progressive patients. Multiplicity was found in 22% and 68% of non-progressive and progressive patients, respectively. A tumour diameter of > 3 cm was found in 34 out of 188 (18%) non-progressive and 6 out of 22 (27%) progressive patients. Recurrent tumours were identified in 59% of progressive and 49% of non-progressive patients.

### Association of miRNA expression and clinical features with progression of NMIBC patients

Among the eight previously reported miRNA [13] tested in the present work, miR-187-3p and miR-25-3p were excluded from further analyses because expression data was not available in >30% of samples (insufficient cDNA was available to perform all qPCR reactions). Supplementary Table 1 shows the fold change differences of the miRNAs between the groups and in relation to the previous study on these miRNAs. Univariate Cox regression analysis including clinical covariates and urinary expression levels of miRNAs identified miR-140-5p, miR-142-3p, miR-18a-3p and miR-92a-3p as predictors of tumour progression (Table 2). Of note, none of the clinical covariates were significantly associated with progression in our cohort of NMIBC. After including the above-mentioned miRNAs in the multivariate stepwise regression analysis, urinary expression of miR-140-5p and miR-92a-3p were found as independent prognostic factors of tumour progression (Table 3).

**Table 2:** Univariate analysis of predictors of tumour progression

Variable	Cut-off level/categories	HR	95% CI	*P* value
Tumour focality	uni vs. multifocal	2.25	0.92-5.52	0.0768
Tumour size	≤3 cm vs. >3 cm	1.69	0.66-4.33	0.2718
BCG treatment	no vs yes	1.29	0.56-2.98	0.5546
Stage^1^	Ta vs. T1	1.06	0.72-1.56	0.7738
Grade^1^	LG vs. HG	1.85	0.75-4.54	0.1792
Concomitant CIS	without CIS vs. CIS	1	0.34-2.95	0.9974
miR-140-5p	−4.4	2.89	1.13-7.39	0.0267*
miR-142-3p	1.01	3.09	1.26-7.6	0.0139*
miR-18a-3p	−7.02	4.40	1.72-11.29	0.0020*
miR-92a-3p	−0.555	5.35	1.81-15.53	0.0025*
miR-125b-5p	−1.74	0.60	0.23-1.53	0.2813
miR-204-5p	−5.15	0.93	0.39-2.21	0.8612

Results of dichotomous clinical variables are expressed considering category placed in second term as the reference category

^1^The grade and stage of the tumours were determined according to WHO criteria [38] and TNM classification [39], respectively.

*Statistically significant

Abbreviations: CIS, Carcinoma *in situ*; LG, Low grade; HG, High grade; BCG, Bacillus Calmette-Guerin; HR, hazard ratio; 95%CI, confidence interval.

**Table 3:** Independent predictors of tumour progression resulting from multivariate stepwise Cox regression analysis

Variable	HR	95% CI	*P* value
miR-140-5p	3.53	1.38-9.06	0.0086*
miR-92a-3p	6.21	2.09-18.45	0.0010*

Abbreviations: HR, hazard ratio; 95%CI, confidence interval

*Statistically significant

### Development of a miRNA-based prognostic classifier for NMIBC patients

**Figure 1:**
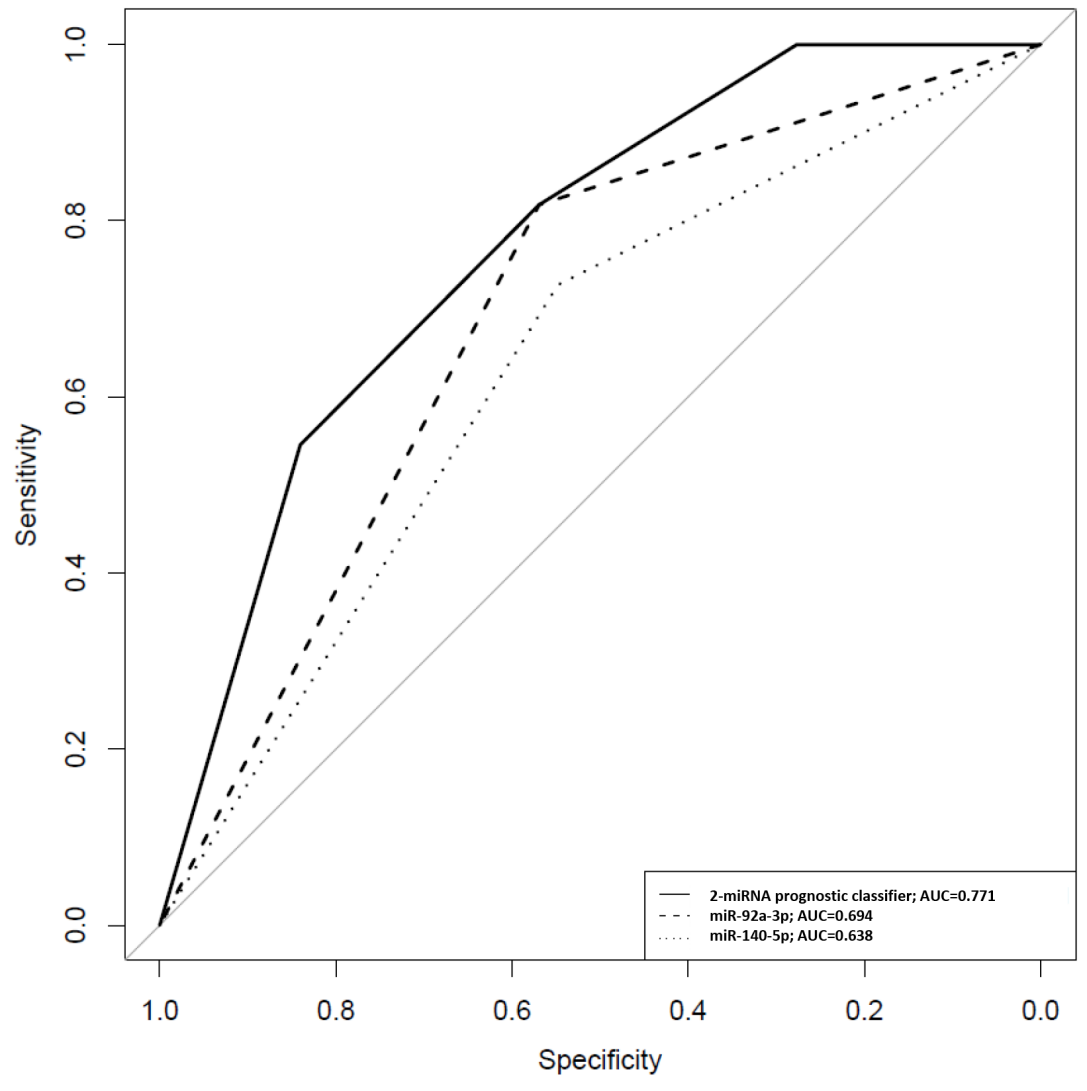
ROC curves for miR-140-5p, miR-92a-3p and the 2-miRNA prognostic classifier based on results obtained by quantitative PCR in urinary samples of NMIBC patients A RS for tumour progression was calculated for each patient according to a mathematical algorithm containing miR-140-5p and miR-92a-3p expression values as described in Patients and methods section. In this equation, miR-140-5p and miR-92a-3p were introduced as dichotomous variables (miR-140-5p expression ≥-4.4=1; <-4.4=0 and miR-92a-3p expression ≥-0.555=1; <-0.555=0). At fixed sensitivity of 80%, specificity for miR-140-5p and miR-92a-3p individually and combined in the 2-miRNA classifier was 32%, 38% and 59%, respectively.

**Figure 2:**
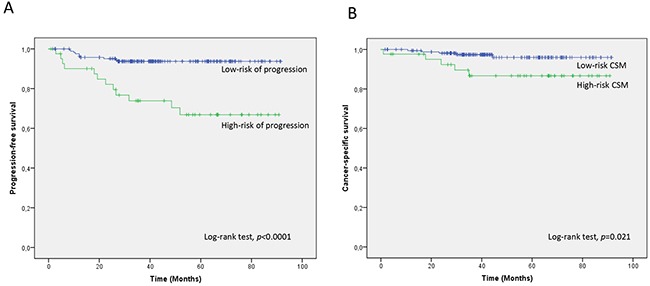
Kaplan-Meier curves for the two-miRNA prognostic classifier showing **A**. time to progression and **B**. time to cancer-specific survival for low-risk (RS<1.62; n=168) and high-risk (RS>1.62; n=42) groups of NMIBC patients. CSM; cancer-specific mortality

**Figure 3:**
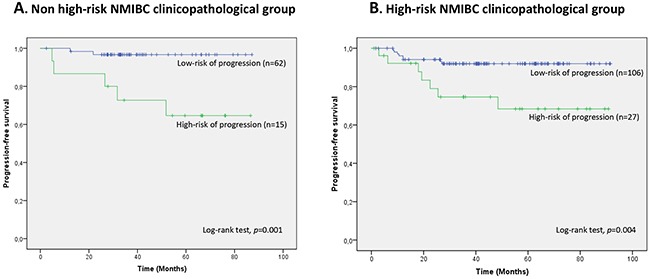
Kaplan-Meier curves for the two-miRNA prognostic classifier showing time to progression in clinicopathological groups of risk **A**. non high-risk NMIBC (n=77) and **B**. high-risk NMIBC (n=133) patients. Patients were divided within each clinicopathological NMIBC risk group according to their RS (RS<1.62, low-risk of progression; RS>1.62, high-risk of progression).

A 2-miRNA prognostic classifier was developed by combining miR-140-5p and miR-92a-3p expression values. A risk score (RS) for tumour progression was calculated for each patient according to a mathematical algorithm containing miR-140-5p and miR-92a-3p expression values. The median value of this RS was −0.206 (range −1.469 to 1.621). Thereafter, a receiver-operating characteristic (ROC) curve analysis of the model (Figure 1) allowed the selection of a cut-off value of 1.62 to classify patients into a high-risk group for tumour progression (20%) and low-risk group for tumour progression (80%) with an accuracy of 81% (55% sensitivity and 84% specificity). Figure 2 depicts Kaplan-Meier curves generated using the selected cut-off point. As shown, RS generated using miRNA expression values was able to discriminate between two groups of NMIBC patients with a highly significant different probability of tumour progression (HR, 5.21; 95%CI, 2.24-12.06; p=0.0001) (Figure 2A). It is important to point out that when patients were stratified according to BC risk groups, the RS continued to discriminate two subgroups of different probability of progression (non-high-risk NMIBC group: HR, 9.59; 95%CI, 1.84-50.09, p=0.007 and high-risk NMIBC group: HR, 3.92; 95%CI, 1.42-10.81, p=0.008) (Figure 3). Moreover, although it did not constitute a primary outcome, accuracy of the RS was also evaluated regarding the probability of being a predictor of cancer-specific survival. Using the same cut-off point, the algorithm was also able to distinguish between two groups with significantly different probabilities of cancer-specific survival (HR 3.88; 95%CI, 1.12-13.44, p=0.033) (Figure 2B).

Finally, robustness of the mathematical model was evaluated by bootstrapping with 1,000 resamples, obtaining a C-index after bootstrapping of 0.7306723.

### Target prediction and functional enrichment of the two miRNA signature

**Figure 4:**
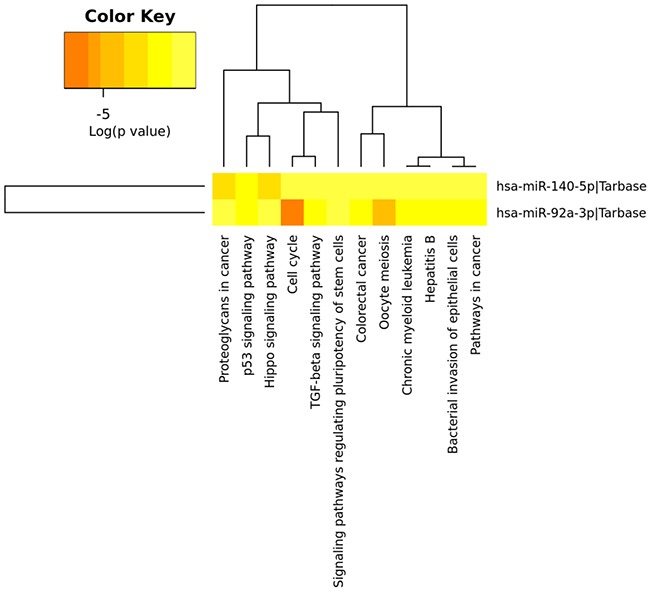
Heatmap of the KEGG pathways enriched in two miRNA target genes Heatmap from intersection of targeted genes (genes targeted by the two miRNAs from the model) is shown. The two miRNAs are involved in multiple common pathways, especially in cancer-specific pathways (DIANA-miRpath computes log_10_ P-values).

The DIANA-miRPath miRNA analysis, by using the 2 miRNAs of the model, showed several statistically significant predicted KEGG terms related to cell cycle, TGF-β signalling pathway, MAPK signalling pathway, Wnt signalling pathway, p53 signalling pathway, and pathways in cancer among others. Consistently, when analysing the data by the gene intersection and union mode, with the two miRNAs targeting the same gene, common terms appear to be significantly enriched such as the p53 signalling pathway, TGF-β signalling pathway, cell cycle, and pathways in cancer (Figure 4 and Supplementary Figure 1).

## DISCUSSION

Prediction of disease progression for patients diagnosed with NMIBC is an important clinical challenge. The most important clinical classifiers for progression currently used, stage T1, HG and presence of CIS [2] where unable to predict progression in our cohort of patients. Neither, the less distinctive clinical prognostic classifiers; recurrence, multiplicity and tumour size, could distinguish progressive from non-progressive patients. One explanation could be that the vast majority of our cohort corresponds to HG NMIBC tumours. This fact could minimize the impact of clinicopathological features on predicting progression, and precisely confirms the critical need for new objective methods to identify those NMIBC patients that are likely to progress. Here, we show that a 2-miRNA urinary signature is able to identify a subgroup of NMIBC patients with high-risk of progression, thus providing additional risk stratification beyond clinicopathological prognosticators.

We and others have previously provided evidence that urinary miRNAs can be used as non-invasive diagnostic biomarkers for BC patients [13–17]. Cell-free urine and serum have also been used as a source of non-invasive diagnostic and prognostic biomarkers of BC [18–20], but as far as we could ascertain, this is the first study that has evaluated urinary cell miRNAs as prognostic biomarkers of BC. The results of this study demonstrate that expression of miR-140-5p and miR-92a-3p in urinary cells independently predicts tumour progression in NMIBC patients. This miRNA signature provides an easy-to-use and reliable algorithm to identify a subgroup of patients with a higher probability of tumour progression and shorter cancer specific survival. An accurate estimation of the progression risk would help to identify the most appropriate therapy for each individual patient. For instance, patients at high risk of progression could benefit from re-TURB, adjuvant therapies, more aggressive treatments or more intensive surveillance. In addition, the 2-miRNA model maintains its prognostic ability when NMIBC patients are stratified according to their clinicopathological risk group, thus reinforcing the validity of our model. Of special interest is the predictive value of the model for progression in the subset of non-high risk NMIBC that are supposed to have low-intermediate risk of progression. Identification of those non-high risk NMIBC patients which are more likely to progress may help to change current treatment schedules for this particular subset of NMIBC patients, intensifying their surveillance schedule.

Regarding the genetic markers included in our algorithm, miR-140-5p has been previously suggested to inhibit the expression of TGF-β and MAPK/ERK signalling pathways in several malignancies [21–25]. In addition, a recent publication reveals that overexpression of miR-140-5p has an inhibitory effect on BC by downregulating the expression of the oncogenic isoform *ΔNp63* of *TP63* [26], a key gene localized in region 3q28 associated with BC risk [27]. In agreement with this data, we previously found a statistically significant downregulation of miR-140-5p in urinary cells from BC patients as well as in high grade BC tumours [13], although this statistically significant downregulation is not seen in the present cohort of NMIBC progressive patients. It is most likely that the different cell types and the variable proportion of tumour cells in the urine of each subject accounts for this lack of statistically significant differences.

Aberrations in miR-92a-3p expression have been previously reported in many cancers [28–30], but to our knowledge, this is the first time miR-92a-3p has been associated with BC. In recent studies, it was observed that overexpression of miR-92a-3p can disrupt PI3K/Akt/mTOR pathway by targeting two negative regulatory factors of this pathway, *PTEN* and *PHLPP* [29, 31]. Different studies have reported that overexpression of miR-92a-3p is involved in the development of metastasis and correlates with survival in different malignancies [32–34]. Our study shows that miR-92a-3p is significantly overexpressed in urinary cells of NMIBC progressing patients which is in concordance with its role as oncomiR.

KEGG pathway analysis corroborates that the 2-miRNA signature is biologically meaningful. It shows that the target genes of the two miRNA take part in many important signalling pathways such as cancer pathways, cell cycle, TGF-β pathway and p53 signalling pathway, among others. Moreover, it has been reported that several of the genes targeted by our miRNAs (e.g. *TP63, PTEN*) [35, 36] play a role in the genetic pathogenesis of BC.

The strengths of this study lie in the fact that it includes a cohort of patients attended in a single centre, homogeneously treated, with prospective data collection and long term follow-up. Furthermore, the use of urine samples to obtain the miRNA prognostic signature may allow the development of a non-invasive bladder cancer prognostic tool with an easy translation into clinical practice. However, some limitations should be acknowledged. First, banked urine samples were analysed, so we cannot calculate the rate of experiment failure due to low cellularity of the sample. Nonetheless, our experience is that the failure rate is low (~5%) in the case of tumour samples [37]. Second, the number of events in the cancer-specific survival analysis is limited. Thus, this part of the results must be taken with care until a validation in a cohort with larger number of events is performed. Next, because of our interest in magnifying the probability to identify reliable prognostic miRNAs, all available samples were included in the evaluation set, thus preventing an independent validation. To overcome this drawback, we strictly ascertained the robustness of the mathematical algorithm by bootstrapping, obtaining a high concordance index. However, the performance of this miRNA signature would be more reliable if validation is performed in an independent external data set. Finally, we have evaluated a limited number of candidate miRNAs previously included in our signatures. Other attractive candidate miRNAs with prognostic value in BC are reported in the literature. Thus, our study rather than establishing a definitive prognostic miRNA expression signature, may contribute to describe prognostic miRNAs which warrant further prospective evaluation in carefully and specifically designed studies.

In summary, our results showed a 2-miRNA urinary signature which significantly predicts progression and cancer-specific survival in NMIBC patients, indicating that it may be a novel potential biomarker for prognosis of NMIBC patients.

## MATERIALS AND METHODS

### Patients and clinical samples

Under Institutional Review Board approval and patients’ informed consent, we prospectively collected freshly voided urine samples from BC patients at Hospital Clínic of Barcelona, starting January 2008. Urines were centrifuged and separated into cellular pellet and supernatant prior to storage at −80°C in a urinary biorepository.

For this study, consecutive available banked cellular pellets from urine samples collected between January 2008 and July 2013 at Hospital Clínic of Barcelona were used for analysis. These included 210 samples from urothelial cell carcinoma of the bladder patients subjected to TURB (Table 1) [38, 39]. The only exclusion criterion was the lack of follow-up after TURB. Tumours were classified according to their clinicopathological risk into two categories: high-risk NMIBC tumours [any of the following: T1 tumours, HG tumours, CIS, and multiple, recurrent, large (>3 cm) Ta low grade tumours (all conditions must present for the last point)] and non high-risk NMIBC tumours (all tumours not defined in the other category). None of the patients included had an upper urinary tract tumour. All patients were followed-up postoperatively following the EAU guidelines [1]. Tumours were considered to be in progression when pathological stage or histological grade increasing and/or distant metastasis or pathological nodes were developed during the follow-up.

### RNA extraction

Around 50-100 ml of urine was collected from each BC patient the day before theTURB. Urine samples were processed as previously described [40], except that collected urines were stored at 4°C and processed within the next 24 hours instead of ice cooled. RNAs from the urinary cell pellets were extracted using TRIzol reagent (Invitrogen, Carlsbad, CA, USA) according to the manufacturer's instructions and quantified with a NanoDrop1000 (NanoDrop Technologies, Wilmington, DE, USA).

### miRNA analysis

A total of 8 key miRNAs (miR-25-3p, miR-18a-3p, miR-92a-3p, miR-140-5p, miR-125b-5p, miR-142-3p, miR-204-5p and miR-187-3p), previously described by our group [13], were selected for examination in urinary samples from NMIBC patients by quantitative PCR by using miRCURY LNA Universal RT microRNA PCR kit (Exiqon, Vedbaek, Denmark), as previously described [13]. Briefly, total RNA (100 ng) containing miRNA was polyadenylated, and cDNA was synthesized using a poly(T) primer with a 3’ degenerate anchor and a 5’ universal tag. Then, cDNA was served as a template for miRNA RT-qPCR amplification with the specific locked nucleic acid (LNA) primers (Supplementary Table 2) and SYBR Green master mix. PCR reactions were carried out using a Light Cycler 480 instrument. The amplification profile was denatured at 95°C for 10 min followed by 45 amplification cycles of 95°C for 10s and 60°C for 1 min. At the end of the PCR cycles, melting curve analyses were performed. miR-103-3p and miR-30c-5p were used as endogenous controls, as previously described [13, 41].

### Statistical analysis

Univariate Cox regression analysis was performed on each covariate to examine its influence on tumour progression and cancer–specific survival. Thereafter, a multivariate forward stepwise Cox regression analysis was performed. After establishing the multivariate model, a RS for the miRNAs of the model was calculated for each patient as the linear combination of the model according to the following RS*=*β*_1_×* miR-140-5p *+*β*_2_×* miR-92a-3p. RS was subjected to a ROC analysis in order to choose the most appropriate threshold for predicting tumour progression. Then, Kaplan-Meier curves for tumour progression and cancer-specific survival were generated using the selected cut-off point and compared according to the log-rank test. Statistical significance was established at p-value of 0.05. R-software and SPSS v23.0 were used for calculations.

Finally, robustness of the mathematical algorithm resulting from the multivariate analysis was evaluated by bootstrapping with 1,000 resamples. For this purpose, validate function from RMS Package was used and the optimism-adjusted estimate of concordance index was computed (http://CRAN.R-project.org/package=rms).

### Pathway enrichment analysis

DIANA-miRPath v3.0 software (http://www.microrna.gr/miRPathv3) [42] using experimentally validated miRNA interactions derived from DIANA-TarBase v7 was used to identify targets of the miRNAs composing the prognostic signature. Subsequent target enrichment analysis was performed in order to discover possible canonical altered pathways using DAVID (https://david.ncifcrf.gov).

## SUPPLEMENTARY MATERIALS FIGURES AND TABLES



## Abbreviations

BC: bladder cancer
BCG: bacillus Calmette-Guerin
CI: confidence interval
CIS/Tis: carcinoma *in situ*
CSM: cancer-specific mortality
HG: high grade
HR: hazard ratio
KEGG: kyoto encyclopedia of genes and genomes
LG: low grade
LNA: locked nucleic acid
miRNA: microRNA
NMIBC: non-muscle invasive bladder cancer
ROC: receiver-operating characteristic
RS: risk score
TURB: transurethral resection of the bladder
Data acquisition: MIT, AC, MC, LG
Data analysis and interpretation: MIT, LM, JJL, MJR, AA, LI
Statistical analysis: JJL, LI
Manuscript preparation: LM
Manuscript review: ALL AUTHORS
