# Supplementary material for: Urinary cell microRNA-based prognostic classifier for non-muscle invasive bladder cancer
Source: Oncotarget. 2017 Feb 14;8(11):18238–47. doi: 10.18632/oncotarget.15315 (PMC5392323; doi:10.18632/oncotarget.15315)
Supplement: Supplementary file 1 [file oncotarget-08-18238-s001.pdf]

Urinary cell microRNA-based prognostic classifier for non-muscle invasive bladder cancer

SUPPLEMENTARY FIGURE AND TABLES

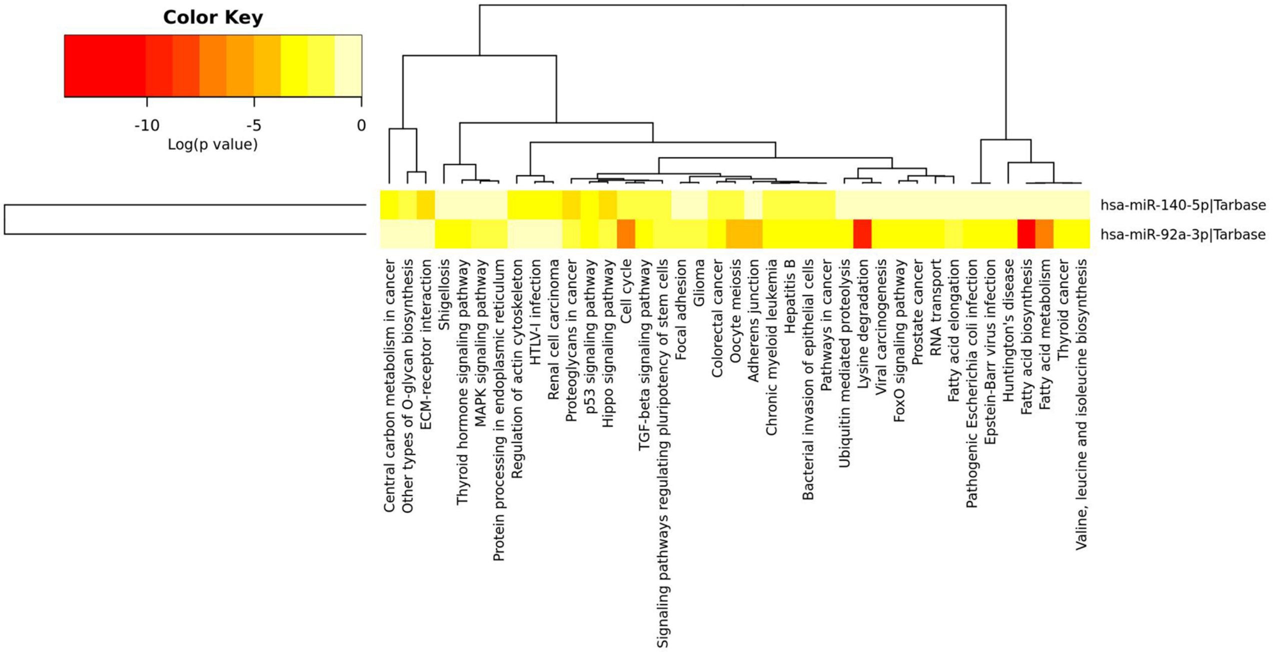

Supplementary Figure 1: Heatmap of the KEGG pathways enriched in two miRNA target genes. Heatmap from union of targeted genes (genes targeted by at least one selected miRNAs) is shown. The two miRNAs from the signature are involved in multiple common pathways, especially in cancer-specific pathways (DIANA-miRpath computes $\log_{10}$ P-values).

Supplementary Table 1: Fold change values for the miRNAs between the groups in the previous and present study

miRNA	Mengual et al, 2013 [13]				Present study	
	Overall diagnostic performance[a]		Prognostic performance[b]		Prognostic performance[c]	
	FCŦ	*P*¥	FCŦ	*P*¥	FCŦ	*P*¥
hsa-miR-18a-3p	2.02	0.162	3.26	0.002	1.64	0.002
hsa-miR-92a-3p	1.98	0.024	3.60	0.001	1.77	0.012
hsa-miR-140-5p	-2.94	0.011	-1.24	0.034	1.67	0.071
hsa-miR-125b-5p	-3.39	0.029	-5.60	0.048	-2.57	0.010
hsa-miR-142-3p	-4.37	0.003	1.16	0.055	2.23	0.073
hsa-miR-204-5p	-2.47	0.323	-6.04	0.379	-2.08	0.128

Ŧ Fold change (FC) differences of the miRNA expression between BC and control urines[a], high and low grade BC tumour urines[b] and urines from progressing and non-progressing patients[c]. FC values were generated from the median expression of the miRNAs from LNA™ real-time RT-qPCR in the groups compared.
¥ Student's t test *P* values.

Supplementary Table 2: Exiqon identification number for the LNA primers used in this study

miRNA	Exiqon ID
hsa-miR-25-3p	204361
hsa-miR-18a-3p	204523
hsa-miR-92a-3p	204258
hsa-miR-140-5p	204540
hsa-miR-142-3p	204291
hsa-miR-125b-5p	205713
hsa-miR-204-5p	206072
hsa-miR-187-3p	204018
